# Primary signet ring cell carcinoma of the uterine cervix: A case report

**DOI:** 10.1097/MD.0000000000026844

**Published:** 2021-08-06

**Authors:** Yeon Hee Kim, Su Jeong Lee, Seon Ui Lee, In Sun Hwang, Kwang Il Yim, Jin Hwi Kim

**Affiliations:** a Department of Obstetrics and Gynecology, Uijeongbu St. Mary's Hospital, College of Medicine, The Catholic University of Korea, Uijeongbu-si, Gyeonggi-do, Korea; b Department of Pathology, Uijeongbu St. Mary's Hospital, College of Medicine, The Catholic University of Korea, Uijeongbu-si, Gyeonggi-do, Korea.

**Keywords:** carcinoma, signet ring cell, survival, treatment, uterine cervical neoplasm

## Abstract

**Rationale:**

Primary signet ring cell carcinoma of the uterine cervix is extremely rare and the clinical characteristics and prognosis are not well known and there are no specific guidelines for treatment.

**Patient concerns:**

A 43-year-old woman was referred to our hospital for abnormal uterine bleeding lasting 1 month.

**Diagnoses:**

Histological examination revealed a signet ring cell carcinoma of the uterine cervix. After evaluation of extragenital origin, the patient was diagnosed International Federation of Gynecology and Obstetrics stage IIIC1 primary signet ring cell carcinoma or the uterine cervix.

**Intervention:**

The patient was prescribed concomitant chemo-radiation followed by intracavitary brachytherapy.

**Outcomes:**

She showed no evidence of disease after treatment but, it recurred after 7 months of last treatment.

**Lessons:**

Different approaches to diagnosis and treatment of this rare disease are needed and molecular pathological studies related to the onset of the disease are required.

## 1 Introduction

Although the incidence of uterine cervical carcinoma has gradually decreased, invasive adenocarcinoma appears to have increased, predominantly in young Korean women.^[1]^ Most adenocarcinomas of the uterine cervix are the endocervical type, and other types are rare. Among them, signet ring cell carcinoma of the uterine cervix is an exceedingly rare type.^[2–4]^ Signet ring cells exhibit round shapes with eccentric nuclei and have abundant mucus granules in the cytoplasm. Signet ring cell carcinoma is defined as mucinous adenocarcinoma with over 50% of cells showing signet ring cell morphology. This type of cancer is considered aggressive and it is typically diagnosed at advanced stages resulting in shorter survival.^[5]^ It is most seen in the stomach (35%–45%), and less frequently in colon, gall bladder, and breast. Because primary signet ring cell carcinoma of the uterine cervix (PSRCC) is extremely rare, the clinical characteristics and prognosis are not well known and there are no specific guidelines for treatment. In this case report, we present an unpredictable clinical course of 43 years old International Federation of Gynecology and Obstetrics (FIGO) stage IIIc PSRCC patient. Additionally, we reviewed previous reports and analyzed distinguishing clinical characteristics of this rare disease.

This case report was prepared following the CARE guidelines. The ethics committee of the Catholic University (UC20ZISI0138) approved the use of anonymized clinical data to be analyzed and published for research purposes. Written informed consent was exempted by the Institutional Review Board because it was a case of a deceased patient.

## 2 Case presentation

**Figure 1:**
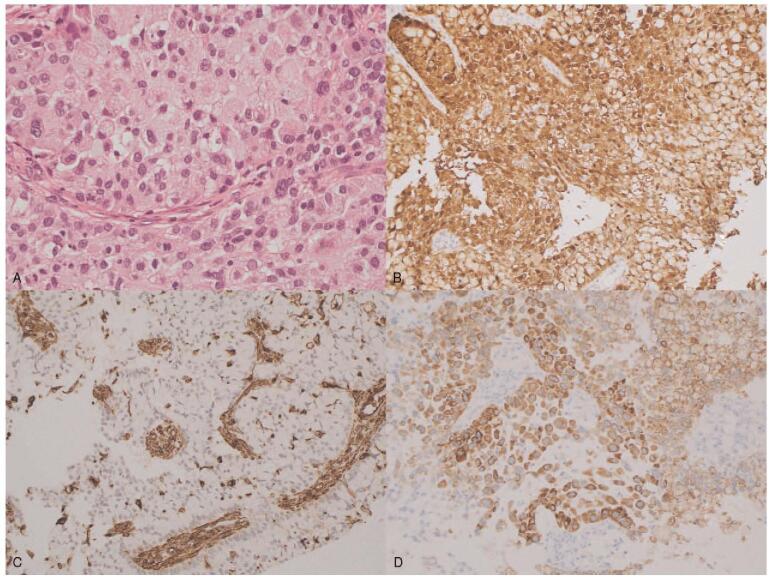
Signet ring cell carcinoma of the uterine cervix. Intracytoplasmic vacuoles are easily seen, some of the nuclei are round while others have a crescentic shape (signet ring morphology) (H&E ×400) (A). The IHC study was conducted to differentiate the primary origin (IHC ×200). The results were P16-positive (B), vimentin-negative (C) and MUC6-positive (D).

**Figure 2:**
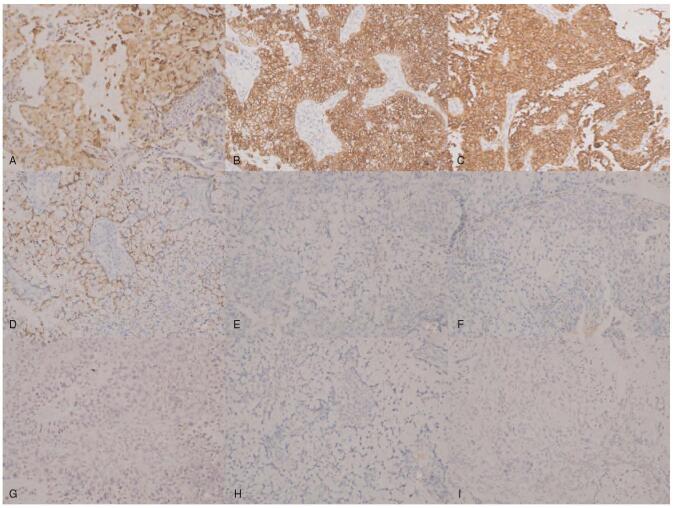
The IHC study was conducted to differentiate the primary origin (IHC ×200). Immunohistochemically, the signet ring cells tested positive for carcinoembryonic antigen (CEA) (A), CK7 (B), MUC 1 (C), 5 (D), and negative for P53 (E), ER (F), CK20 (G), MUC 2 (H), CDX-2 (I).

**Figure 3:**
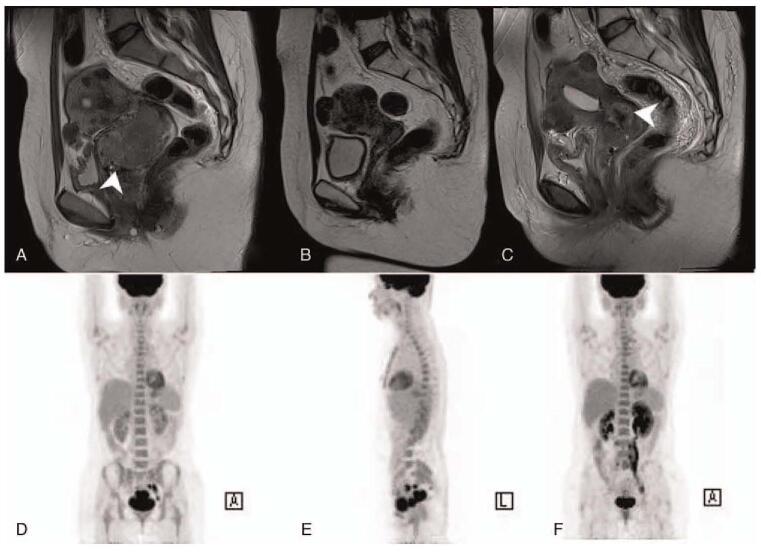
MRI T2 weighted sagittal image shows about 6.8 cm sized mass in cervical canal, induration extended to right parametrium (arrow) (A). Much regression of known cervical cancer after treatment (B). Small nodular lesions in the endocervical canal (arrow) (C). PET-CT show hypermetabolic mass, r/o cervical cancer (max SUV 10.6) and hypermetabolic nodule, in Lt. obturator, Lt. external iliac, r/o metastatic LN (D, E). Hypermetabolic cervical cancer with progression of metastatic LN (F).

The patient was a 43-year-old, gravida 0 woman who presented with abnormal uterine bleeding over the previous 4 months. Pelvic examination revealed a bulky cervical mass with irregular margin and easy-touch bleeding. Cytologic examination of cervical smears revealed conglomeration of atypical cells with intracellular mucinous vacuoles. Human papilloma virus (HPV) genotyping assays were performed using exfoliated cervical cells by polymerase chain reaction and showed HPV18 positivity. Cervical punch biopsy revealed a poorly differentiated adenocarcinoma with a signet ring cell pattern. On high power, intracytoplasmic vacuoles are easily seen, and some of the nuclei are round and others have a crescent shape (Fig. 1). Immunohistochemically, the signet ring cells tested positive for p16 and carcinoembryonic antigen (CEA), CK7, MUC 1, 5, 6, and negative for P53, ER, Vimentin, CK20, MUC 2, CDX-2 (Fig. 2). The elevated tumor marker levels were: carbohydrate antigen 125 (CA-125) 510 U/ml, CEA 100.8 ng/ml, and squamous cell carcinoma-related antigen (SCC) 9.50 ng/ml. Magnetic resonance imaging (MRI) and positron emission tomography (PET) images showed a 6.8 cm sized cervical mass with diffusion restriction which invaded the right parametrium and metastatic lymph nodes in the left obturator, external iliac, bilateral common iliac, and presacral areas (Fig. 3A, D, E). Gastroscopy, colonoscopy and breast sonography were all negative; therefore, we could rule out an extragenital primary tumor. The patient underwent single port para-aortic lymph node dissection for surgical diagnosis and ovarian transposition to preserve the function of both ovaries. Because there was no paraaortic node metastasis, the case was finally diagnosed as PSRCC with the FIGO stage IIIC1. She was prescribed concurrent chemo-radiation (50.4 Gy of pelvic-external radiation and 40 mg/m^2^ of weekly intravenous cisplatin) followed by intracavitary high-dose rate brachytherapy to a total dose of 27.5 Gy. She was followed up at 3 months after chemoradiation where her cervical smear revealed no evidence of malignancy, MRI showed regression of cervical cancer and of the metastatic lymph nodes in external iliac and presacral areas (Fig. 3B). Tumor markers were decreased to within normal limits (CA-125 13.87 U/ml, CEA 1.3 ng/ml, and SCC 2.08 ng/ml).

Seven months after her last treatment, she complained of hematuria and the tumor markers were beginning to elevate (CA-125 176.50 U/ml, CEA 30.0 ng/ml, and SCC 5.94 ng/ml). Cervical cytology examination showed signet ring cell carcinoma. MRI and PET images demonstrated progression of metastatic lymph nodes in the bilateral obturator, left external, bilateral common iliac, left paraaortic, aortocaval and retrocaval areas and left hydronephrosis (Fig. 3C, F). Although 6 cycles of cisplatin-paclitaxel-bevacizumab chemotherapy were administered, the disease progressed, and she refused further treatment strategy. Unfortunately, she expired due to thrombocytopenia at 15 months after diagnosis. We obtained the patient's medical records and reviewed the related literature. Because the patient expired, informed written consent was waived. This study was approved by the Institutional Review Board of Uijeong-bu St. Mary's Hospital (2019-2407-0001) and was conducted in accordance with the principles of the Helsinki Declaration II.

## 3 Discussion

Mucinous type adenocarcinoma of the uterine cervix includes endocervical, intestinal, signet ring cell (SRC), minimal deviation, and villoglandular types. Although the exact pathogenesis of the SRC is unknown, the ErbB2/ErbB3 pathway is important for the mechanism behind this malignant phenotype. Both the MEK1 and p38 MAP kinase pathways contribute to dissociation of the cells. Secretion of Muc4, which activates ErbB2, is enhanced due to the activation of PI3K. This creates an activation loop of ErbB2/ErbB3–Muc4–ErbB2/ErbB3. Overall, if there is a trigger for activation of these pathways, and if cells are basically transformed, signet ring cell carcinomas will be formed.^[6]^ When we detect signet ring cell carcinoma in uterine cervix it is important to distinguish primary from metastatic lesion. The clinical characteristics and survival rates of PSRCC are not well known. There are several reviews in the literature, but the information provided is variable. We reviewed and organized original reports and then reclassified the clinical results by converting them to the 2018 FIGO stage (Table 1). Twenty five cases had the patient's clinical information and 2 cases reported only pathologic findings of paraffin blocks. The age of diagnosis of PSRCC ranged from 29 to 80 years and the mean age was 47.9 years (median age: 46 years). Most patients were multiparous (13/15). The most common symptoms included abnormal uterine bleeding (7/23) and postcoital bleeding (6/23). Only 4 of the 10 cases were diagnosed with poorly differentiated adenocarcinoma from a pap smear. The cytologic diagnosis of adenocarcinoma is more difficult than SCC because its origin is endocervical canal. The cytologic diagnosis of PSRCC is also difficult, since the presence of signet ring cells on smear may also be due to a metastatic carcinoma to the cervix or to a gastrointestinal carcinoma with ascites without cervical involvement.^[7]^

**Table 1:** Summary of reported cases with primary signet ring cell carcinoma of the uterine cervix.

Author	Age	Para	Menopause	Symptom	Pap	HPV	SRC components	PET	Original Stage	New Stage	Treatment	Outcome
Moll, 1990^[12]^	50	NA	NA	PCB	NA	NA	Predominent	NA	III	NA	S+R	DOD10M
Mayorga, 1997^[13]^	68	2	Yes	PCB	NA	NA	Predominent	NA	IB	IB2	C+S	NED35M
	74	2	Yes	PMB	NA	NA	Predominent	NA	IB	IB2	S	NED25M
Haswani, 1998^[14]^	33	NA	NA	ASx	NA	18	Predominent	NA	IIIB	NA	R+C	DOD10M
	38	NA	NA	PCB	NA	NA	Predominent	NA	IB	NA	S+R	NED18M
Cardosi, 1999^[15]^	53	NA	NA	NA	NA	NA	NA	NA	IB	NA	S+CCRT	NED6M
Moritani, 2004^[16]^	29	NA	No	AUB	Atypical	Negative	Focal	NA	IIIB	IIIC1	S+C	NED6M
Suárez-Pẽnaranda, 2007^[17]^	80	Multi	Yes	VD	NA	NA	Predominent	Cx	IIIB	IIIB	C+CCRT	DOD19M
Insabato, 2007^[10]^	46	NA	NA	AUB	NA	NA	Pure	NA	IB1	NA	S	NED36M
McCluggage, 2008^[18]^	NA	NA	NA	NA	NA	NA	Focal	NA	NA	NA	NA	NA
(Paraffin block)	NA	NA	NA	NA	NA	NA	Focal	NA	NA	NA	NA	NA
Veras, 2009^[19]^	36	Multi	No	TE	Negative	Positive	Focal	NA	IVB	IVB	C	DOD7W
	43	NA	NA	LAP	NA	18	Focal	NA	IVB	IVB	C	DOD2M
Lowery, 2009^[11]^	60	Multi	Yes	PMB	Inadequate	NA	NA	NA	IB1	NA	R+S	NED10Y
Balci, 2010^[4]^	53	3	Yes	PMB	Adenoca	18	Predominent	NA	IIB	IIIC1	S	NA
Yoon, 2011^[20]^	47	4	NA	PCB	Adenoca	Negative	Predominent	Cx	IB1	IB1	S	DOD6M
Giordano, 2012^[21]^	45	NA	NA	AUB	NA	18	Focal	NA	IIB	IIIC1	S	NA
Kaidar-Person, 2013^[22]^	37	4	No	PCB	NA	NA	NA	Cx+PN	IIB	IIIC1	R+S	NED4M
Washimi, 2015^[3]^	31	2	No	AUB	Atypical	18	Pure	Cx	IIA	IIIC1	S+C	NED41M
Cracchiolo, 2016^[23]^	64	NA	Yes	Abd pain	Adenoca	NA	NA	Cx	IVB	IVB	Palliative	DOD3M
Sal, 2016^[24]^	48	3	No	PCB	NA	18	Predominent	Cx	IB1	IB2	S	NED18M
Doghri, 2017^[25]^	48	5	No	AUB	NA	NA	NA	NA	IVB	IVB	None	DOD3M
Wang, 2018^[26]^	48	NA	Yes	PMB	Negative	NA	NA	NA	IVB	IVB	S+C	SD13M
Hamada, 2019^[2]^	40	Multi	No	AUB	NA	NA	Predominent	Cx	IB2	IIIC1	C+S	AWD29M
	44	Multi	No	NA	NA	NA	Focal	Cx	IB1	IB2	S	NED15M
Kawai, 2019^[27]^	40	0	No	ASx	Atypical	16	NA	NA	IB1	IB2	S+C	NED33M
Present case	43	0	No	AUB	Atypical	18	Predominent	Cx+PN	IIIC1	IIIC1	CCRT+C	DOD15M

The primary cervical origin could be supported by the presence of HPV DNA. HPV infection contributes to the onset and progression of cervical cancer.^[8]^ HPV tests were conducted on 11 cases and were positive in 9 cases. Out of the 9 HPV positive cases, 8 were identified by HPV typing of which, 7 were HPV 18-positive and one was HPV 16-positive. HPV type 18 is often seen in cervical adenocarcinomas.^[9]^ Combining the results so far, PSRCC and HPV 18 infections are strongly related. Although the clinical significance of the SRC components is unclear, of the 21 cases in which the ratio of SRC components was identified, 7 cases were focal, and 14 cases were predominant. Increased uptake of FDG in both primary and metastatic lesions have been found in all 8 previously reported PSRCC cases that had undergone PET/CT. In our case, it was possible to detect pelvic node metastasis, and the paraaortic node was negative and matched with the surgical result. Therefore, PET/CT can also be helpful for detecting an extragenital lesion and choosing a surgical treatment. Of course, in all cases, extragenital evaluations such as gastroscopy, colonoscopy and breast sonography were implemented to diagnose a primary lesion.

**Figure 4:**
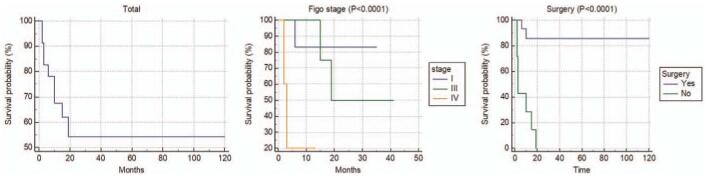
Survival curves (Kaplan–Meier survival curves) for patients with primary signet ring cell carcinoma of the uterine cervix.

Of the 18 cases that could be converted to the 2019 FIGO stage, 6 were stage I, 7 were Stage III, and the remaining 5 cases were stage IV. Five cases of stage I or II on the FIGO stage at the time of publication were upgraded to stage III on the 2019 FIGO due to lymph node metastasis at diagnosis. Advanced stage with lymph node metastasis at the time of diagnosis is believed to be related to the poor prognosis of this disease. It is difficult to predict survival and prognosis because of the paucity of previous case reports. However, this suggests that an advanced stage of disease is especially aggressive and earlier stage disease shows better prognosis.^[10,11]^ The median survival of the 23 cases that could be analyzed for survival was 25 months. Among age, SRC component, FIGO stage, and surgical treatment, FIGO stage and surgical treatment were associated with survival (Fig. 4). The survival rate was significantly higher in the group that underwent treatment including surgery, which was consistent with previous studies.^[2]^ Chemotherapy was performed on our patient due to the rapid recurrence during progress observation after radiation treatment, but there was no improvement. Even in this case, if general condition were operable, a palliative operation could have been considered.

Various immunohistochemical studies were performed in most of the previous cases (Table 2). P16, which indicates HPV infection, was positive in 12 of the 13 cases. CEA, which indicated adenocarcinoma, was positive in 10 of the 11 cases. Vimentin, which was used to exclude endometrial origin, was negative in all 5 cases, while estrogen receptor/progesterone receptor (ER/PR) was positive in one of 14 cases. Ten of 11 cases showed a positive reaction for CK7, associated with high-grade cervical lesions, and 10 of 12 cases showed a negative reaction for CK20, which is associated with colorectal lesions. Most of the other colorectal and gastric cancer markers such as CDX2, MUC1, MUC2, and MUC6 were also negative. In the present case, the immunohistochemical staining pattern of CEA, p16, CK7, and ER showed findings consistent with a primary cervical neoplasm. However, while MUC6 was shown to be negative in all of 4 cases that were stained, for the first time, our case showed positive. This finding might be considered as unexpected, since MUC6 is a gastric marker. Immunohistochemical studies do not provide a definitive clinical diagnosis of primary signet cell cervical cancer. Based on conflicting results, immunohistochemical studies could be limited role to rejecting or proving the diagnosis of primary signet cell cervical cancer.

**Table 2:** Immunohistochemical studies performed in the previous cases.

Author	CEA	CK22	P16	Vimentin	ER	PR	CK7	CK20	CDX2	MUC6	MUC5AC	MUC1	MUC2	Muscarmine	PAS	AB	P63
Moll,1990^[12]^																	
Mayorga,1997^[13]^	+	+												+	+	+	
	+	+												+	+	+	
Haswani, 1998^[14]^					−												
					−	−											
Cardosi, 1999^[15]^					+												
Moritani, 2004^[16]^		+			−	−				−	+		−		+	+	−
Suárez-Pẽnaranda, 2007^[17]^	+	+		−	−	−											
Insabato, 2007^[10]^																	
McCluggage, 2017^[18]^	+		+				+	−	−								
	−		+				+	−	−								
Veras, 2009^[19]^			+		−	−	+	−	−								
			+		−	−	+	−	−								
Lowery, 2009^[11]^																	
Balci, 2010^[4]^	+		+	−	−	−	+	+	−		+	+	−		+	+	
Yoon, 2011^[20]^																	
Giordano, 2012^[21]^	+		+	−	−	−	+								+	+	
Kaidar-Person^[22]^																	
Washimi, 2015^[3]^	+		−		−	−	+	−	+	−	−	−	+		+	+	
Cracchiolo, 2016^[23]^	+		+		−	−		−	−		+				+		
Sal, 2016^[24]^			+		−	−		−	+			+	+				
Doghri, 2017^[25]^	+		+	−	−	−	+	−									
Wang, 2018^[26]^			+				−	+		−							
Kawai, 2019^[27]^			+				+	−	−	−	+		−				
Present case	+		+	−	−		+	−	−	+	+	+	−		+	+	

Author	P53	Chromogranin A	Synaptophysin	CD56	PGP 9.5	s-100	GCDFP 15	Mammoglobin	TTF-1	Rb	SMA	ca125	beta catenin	e-cadherin
Moll,1990^[12]^														
Mayorga,1997^[13]^														
Haswani, 1998^[14]^														
Cardosi, 1999^[15]^														
Moritani, 2004^[16]^														
Suárez-Pẽnaranda, 2007^[17]^		+	+											
Insabato, 2007^[10]^														
McCluggage, 2017^[18]^														
Veras, 2009^[19]^														
Lowery, 2009^[11]^														
Balci, 2010^[4]^		−	−	−	−		−	−	−					
Yoon, 2011^[20]^	+									+				
Giordano, 2012^[21]^														
Kaidar-Person^[22]^		−	−											
Washimi, 2015^[3]^	−	−					−	−	−					
Cracchiolo, 2016^[23]^			−			−	−				−			
Sal, 2016^[24]^		−	−											
Doghri, 2017^[25]^			−											
Wang, 2018^[26]^		−	−	−										
Kawai, 2019^[27]^	−						−		−			+	−	−
Present case	−													

In this case, patient was diagnosed FIGO stage IIIC PSRCC, and rapidly worsened and turned into expire even though initial response to the standard radiation treatment was good. PSRCC might have different therapeutic response and prognosis. Therefore, different approaches to diagnosis and treatment of the disease are needed and molecular pathological studies related to the onset of the disease are required.

## Author contributions

**Conceptualization:** Jin Hwi Kim.

**Data curation:** Yeon Hee Kim, Su Jeong Lee, Seon Ui Lee, In Sun Hwang, Kwang Il Yim, Jin Hwi Kim.

**Formal analysis:** Yeon Hee Kim, In Sun Hwang, Kwang Il Yim, Jin Hwi Kim.

**Investigation:** Jin Hwi Kim.

**Methodology:** Yeon Hee Kim, Jin Hwi Kim.

**Resources:** Yeon Hee Kim, Su Jeong Lee, Seon Ui Lee, Kwang Il Yim, Jin Hwi Kim.

**Software:** Jin Hwi Kim.

**Supervision:** Kwang Il Yim, Jin Hwi Kim.

**Validation:** Kwang Il Yim.

**Visualization:** Kwang Il Yim.

**Writing – original draft:** Seon Ui Lee, Jin Hwi Kim.

**Writing – review & editing:** Yeon Hee Kim, Jin Hwi Kim.
